# Interplay between dietary antioxidants and oxidative/nitrosative stress in stage III periodontitis: a preliminary quasi-experimental study in a Mexican population

**DOI:** 10.3389/froh.2026.1779098

**Published:** 2026-05-07

**Authors:** Miguel A. Robledo-Valdez, Alondra Ruiz-Gutierrez, Mariana Romero-Padilla, Fermin Paul Pacheco-Moises, Fabiola Marquez-Sandoval, Carmen de la Rocha, Adolfo Daniel Rodriguez-Carrizalez, Lucrecia Carrera-Quintanar

**Affiliations:** 1 Departamento de Alimentación y Nutrición, Doctorado en Ciencias de la Nutrición Traslacional (DCNT), Centro Universitario de Ciencias de la Salud (CUCS), Universidad de Guadalajara (UdeG), Guadalajara, Jalisco, Mexico; 2 Departamento de Clínicas Odontológicas Integrales, Especialidad en Periodoncia, Centro Universitario de Ciencias de la Salud (CUCS), Universidad de Guadalajara (UdeG), Guadalajara, Jalisco, Mexico; 3 Departamento de Química, Centro Universitario de Ciencias Exactas e Ingenierias (CUCEI), Universidad de Guadalajara, Guadalajara, Jalisco, Mexico; 4 Departamento de Clínicas de la Reproducción Humana Crecimiento y Desarrollo Infantil, Instituo de Investigación en Cáncer en la Infancia y Adolescencia (INICIA), Centro Universitario de Ciencias de la Salud (CUCS), Universidad de Guadalajara (UdeG), Guadalajara, Jalisco, Mexico; 5 Departamento de Fisiología, Instituto de Terapéutica Experimental y Clínica (INTEC), Centro Universitario de Ciencias de la Salud (CUCS), Universidad de Guadalajara (UdeG), Guadalajara, Jalisco, Mexico

**Keywords:** antioxidants, diet, oxidative stress, periodonditis, periodontitis

## Abstract

**Introduction:**

Stage III periodontitis is a severe inflammatory disease characterized by significant tissue destruction. While Non-Surgical Periodontal Therapy (NSPT) is the gold standard, evidence suggests it may only or partially address of systemic oxidative imbalance. This study aimed to investigate the interplay between habitual dietary antioxidants, NSPT, and oxidative/nitrosative status in Mexican patients, a population with unique dietary patterns that may fuel disease pathways.

**Methods:**

A preliminary quasi-experimental study was conducted with 24 adults (11 with periodontitis; 13 healthy controls). Clinical examinations were performed by a calibrated periodontist. Habitual diet was assessed at baseline using a validated SFFQ. Salivary and plasma levels of Total Antioxidant Capacity (TAC), protein carbonyls (CARBO), lipid peroxidation (LPO), and nitrate/nitrite were measured.

**Results:**

The periodontitis group exhibited significantly higher energy and lower polyunsaturated fatty acid (PUFA) intake (*p* < 0.05). A pivotal finding was the metabolic hyper-reactivity in diseased patients (30 significant nutrient-biomarker correlations vs. 5 in controls). Following NSPT, clinical parameters and plasma TAC improved (*p* < 0.05); however, salivary MDA+4-HDA remained at 0.94 μM/L—above the healthy threshold of 0.77 μM/L, consistent with a non-significant reduction in the Plaque/Calculus Index(*p* = 0.059).

**Conclusions:**

Our findings suggest that NSPT alone is insufficient to fully restore physiological oxidative homeostasis. The persistence of altered oxidative markers, linked to residual biofilm and dietary imbalances, supports the integration of targeted nutritional strategies as a necessary adjunct to achieve complete biological recovery in Stage III periodontitis; further large-scale studies are warranted to validate these results.

## 1 Introduction

In 2021, the global burden of disease reported 1 billion people affected by severe periodontitis (1066.95 million; 95% UI: 896.55-1234.84) (1). In Mexico, the Epidemiological Surveillance System (SIVEPAB) reports the community periodontal index for a population of 52,938 patients, of whom 60% presented signs of periodontal disease and 1% signs of advanced periodontitis (2).

Periodontitis is defined as an inflammatory infectious disease that begins with the presence of pathogenic microorganisms in dental plaque. In 2017, the European Federation of Periodontology and the American Association of Periodontology proposed the Classification of Periodontal and Peri-implant Diseases and Conditions. One of the objectives of this classification is to provide case definitions that facilitate a more accurate study of affected populations. In this context, stage III periodontitis is considered a severe form that generates destruction of periodontal tissue, affecting the middle and apical thirds of dental support structures (3).

The host's defense response against these pathogens involves neutrophil production of reactive oxygen species (ROS). Although the response is focused on eradicating bacteria, they are also capable of tissue destruction (4). ROS can cause damage via multiple mechanisms, including DNA damage, lipid peroxidation, protein damage, impairing their function, and the stimulation of proinflammatory cytokines. At sites of chronic inﬂammation, there is also an overproduction of nitric oxide, often due to increased expression of inducible nitric oxide synthase (iNOS), which produces reactive nitrogen species (RNS). This process is called nitrosative stress (5, 6).

Due to their high reactivity, ROS and RNS have a very short half-life, making them difficult to detect directly. However, measuring the secondary metabolites resulting from the damage they cause can be highly useful in assessing their impact on periodontitis. Some of these biomarkers include total antioxidant capacity (TAC), malondialdehyde (MDA) and 4-hydroxyalkenals (4-HDA) as lipid peroxidation byproducts, nitrate/nitrite as nitrosative stress indicators, and protein carbonyls (CARBO). Variations in the concentrations of these biomarkers, in both saliva and blood, have been shown to correlate with periodontitis in general, accounting for the disease stage. These findings have been observed separately, but the interaction between them remains unknown, as does the manner in which it occurs in this population (5, 7). Even though non-surgical periodontal therapy (NSPT) is the gold standard for biofilm control, evidence suggests that it may offer only partial control of systemic oxidative imbalance, leaving biomarkers such as malondialdehyde (MDA) elevated even after clinical improvement (8, 9).

Beyond its high prevalence in Mexico, where nearly 60% of the population shows signs of periodontal disease, this population represents a critical study model due to its unique dietary landscape. Mexican dietary patterns, often characterized by excessive caloric intake and potential imbalances in protective nutrients, may directly fuel oxidative and nitrosative pathways that drive tissue destruction in periodontitis (1, 2). Given that high energy consumption generates mitochondrial substrates that accelerate oxidative processes, the Mexican population provides a relevant context to study how specific nutritional deficiencies, such as lower polyunsaturated fatty acid (PUFA) intake, exacerbate the inflammatory burden of Stage III periodontitis (10–12).

Furthermore, it has been established that certain dietary elements can either promote or prevent nitrosative and oxidative stress. High intake of nutritional energy, from carbohydrates or lipids generates substrates for mitochondrial respiration, a highly oxidative process. On the contrary, a healthy diet that contains dietary ﬁber, unsaturated fatty acids like monounsaturated fatty acids (MUFA) and n-3 polyunsaturated fatty acid (n-3 PUFA), protein, vitamins, minerals, and other health-promoting components exhibit antioxidant ability, thereby reducing oxidative stress (13). Dietary TAC has been correlated with plasma TAC in young adults, but not in adults with periodontitis (14).

Despite these links, the specific interplay between dietary antioxidant components and salivary oxidative/nitrosative markers in Mexican patients remains undescribed. We hypothesize that NSPT alone is insufficient to restore physiological oxidative homeostasis without addressing the underlying nutritional contributors. Therefore, this study aims to evaluate the relationship between diet and NSPT with respect to local and systemic markers to determine if nutritional intervention is a necessary adjunct to achieve complete biological recovery in Stage III periodontitis.

## 2 Materials and methods

### 2.1 Study design

A preliminary quasi-experimental study was conducted. This design was selected because the primary objective involved comparing participants with a pre-existing pathological condition (Stage III periodontitis) against a healthy control group, which precludes the use of random allocation for the disease status. The study protocol was conducted in accordance with the Declaration of Helsinki Guidelines and was approved by the ethics, research, and biosafety committees of the University Center of Health Sciences at the University of Guadalajara (register number 22-75; CI 05722 CUCS-UdeG). All participants provided written informed consent. This study was performed following the CONSORT Statement for Randomized Trials of Nonpharmacologic Treatments: A 2017 Update and a CONSORT Extension for Nonpharmacologic Trial Abstracts (Supplementary Materials, Table S1) (15).

### 2.2 Study population

Patients with periodontitis stage III according to the classification established by the World Workshop 2017, who attended consultation in the Specialty of Periodontics of the Integral Dental Clinics of the University Center of Health Sciences from the University of Guadalajara, Mexico, during 2022-2023.

The eligibility criteria included men and women aged 30 to 70 years, along with complete clinical records that provided information on sociodemographic, anthropometric, clinical, salivary, and blood samples of the subjects. Participants included denying having a previous diagnosis of diabetes, dyslipidemia, hypertension, autoimmune diseases, among other conditions, as well as denying the use of related medications.

**Figure 1:**
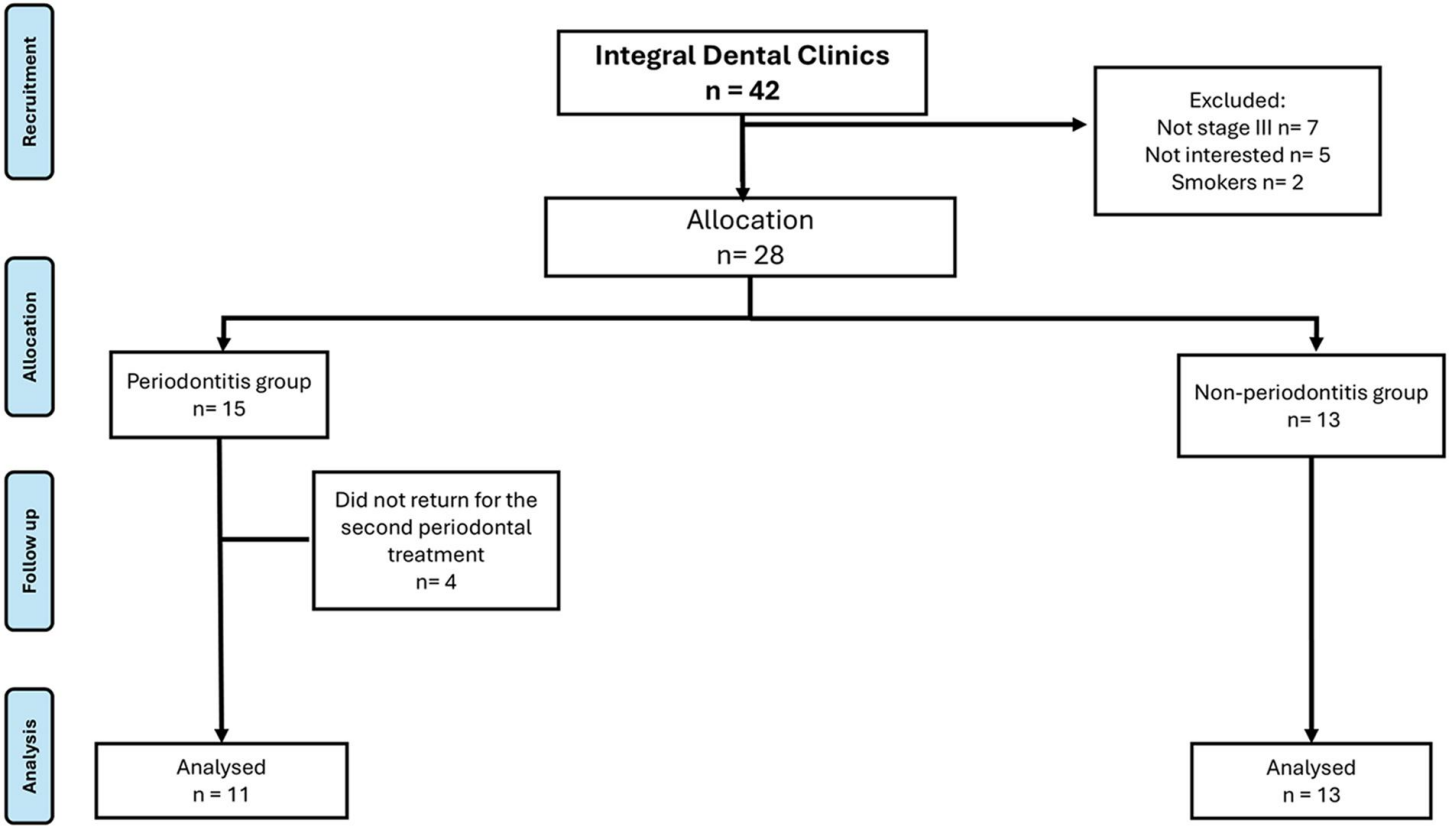
CONSORT flowchart of the study participants.

The sample size was calculated according to the study by Nisha et al. We considered a mean difference of 38 units of saliva total antioxidant capacity. A sample size of 22 patients in total (11 per group) was used to achieve a power of 80% and a level of significance of 5%, and 20% of subjects lost to follow-up (16). Given the final analyzed cohort of 24 participants, this work is explicitly defined as a preliminary pilot study. While the sample size is limited, the study focuses on high-precision, interdisciplinary measurements (periodontal, nutritional, and biochemical) conducted by a specialized team to minimize confounding factors, a depth of detail often prioritized in high-quality small-sample medical research. Recruitment was conducted via non-probabilistic sampling at the Specialty of Periodontics between 2022 and 2023. Our sampling method was non-probabilistic. The participant selection process is detailed in the CONSORT flowchart (Figure 1), which specifies the reasons for exclusion, such as a smoking habit or recent use of antibiotics, to ensure methodological rigor and minimize confounding variables.

#### 2.2.1 Group with periodontitis

For stage III, according to the 2017 Workshop, the following severity was considered: at least two non-contiguous clinical attachment loss (CAL) sites ≥5 mm (except third molars and distal second molars), radiographic bone loss extending to the middle third of the root and beyond, and tooth loss of ≤4 teeth due to periodontitis. Regarding local complexity: Probing depth ≥6 mm, vertical bone loss ≥3 mm, furcation involvement, class II or III, and moderate ridge defect. Bleeding on probing (BOP) ≥ 10% and the need for non-surgical periodontal therapy (NSPT) were also considered (17). In turn, the grades were evaluated through indirect evidence of progression, using the percentage of radiographic bone loss in relation to the patient's age. For this purpose, the tooth with the most significant radiographic bone loss was selected. If the % bone loss/patient's age values ​​were <0.25, grade A was assigned. If they were between 0.25 and 1, grade B was assigned. If they were >1, grade C.

#### 2.2.2 Group without periodontitis

Periodontally healthy patients, without any metabolic disease self-reported or diagnosed by their treating physician, non-CAL, non-radiographic evidence of periodontal bone loss, PPD ≤ 3 mm, and ≤ 10% of BOP (18).

#### 2.2.3 Non-inclusion criteria (both groups)

Trauma-induced gingival recession, cervical caries, history of bleeding diathesis, pregnant or lactating women, history of any systemic disease by self-report or use of medication prescribed by a physician, having received periodontal treatment in the previous 6 months, use of antibiotics, and smoking habit.

### 2.3 Assessment of periodontal clinical variables

Each patient underwent a periodontal medical history: reason for consultation, family, medical, and dental history, oral hygiene habits, and medications, as well as tobacco, alcohol, and drug use. A complete periapical radiographic study was also taken. The clinical periodontal examination was conducted on the teeth present in each participant's mouth; clinical parameters, including CAL, PPD, % BOP, and % Plaque or Dental Calculus (PoC), were recorded in a periodontogram. To validate the variability and reproducibility of periodontal clinical measurements, intra-examiner calibration tests were performed by the periodontist, yielding an average variability of 0.06 and an average reproducibility of 93.7%, thereby qualifying the periodontist as a suitable clinical evaluator (19). The examination was performed with a North Carolina-type periodontal probe (Hu-Friedy UNC CP-15, Chicago, IL, USA), a Nabers probe (Hu-Friedy N6 Coded Nabers Probe), and a mouth mirror. For each tooth, six measurements were recorded in millimeters (mesial, middle, and distal in buccal and lingual) of PPD and CAL. The results were expressed as averages. The PoC was recorded during clinical assessment, depending on the presence or absence of plaque or calculus at the gingival margin. Meanwhile, BOP was recorded during periodontal probing (assuming a light probing pressure of 0.2 to 0.25 N) as present or absent. PoC and BOP were recorded at six sites per tooth; the results were expressed as percentages. Patients were classified according to the 2017 Workshop of the American Association of Periodontology (AAP) and the European Federation of Periodontology (EFP) into periodontally healthy subjects and those with stage III and higher degrees of disease (3).

### 2.4 Phase I periodontal therapy

Patients received periodontal treatment, including professional mechanical plaque removal and subgingival instrumentation at all sites requiring this (previously referred to as NSPT). Both ultrasonic and manual instrumentation were used throughout the study, which were previously reported to lead to similar microbiological and clinical outcomes. An experienced periodontist conducted the periodontal treatment in two visits (quadrant 1 and 4 during visit 1 and quadrant 2 and 3 during visit 2). Antibiotics or chlorhexidine and other antiseptics were not used or prescribed during the study; this was intentional to evaluate the pure effect of mechanical instrumentation and dietary components on the clinical and oxidative/nitrosative biomarkers. As part of the periodontal treatment, patients received comprehensive instructions and regular monitoring of their oral care, including correct tooth brushing and interdental cleaning, provided by the periodontist. Only patients with periodntitis received Phase I of periodontal therapy after baseline sample collection and clinical measurements. No additional instructions were given to modify other aspects of their lifestyles. Full-mouth periodontal parameters were assessed before treatment (baseline) and again 70 days after the treatment. These included PPD, CAL, BOP, and PoC.

### 2.5 Dietary assessment

To evaluate food intake, a Semi-Quantitative Food Frequency Questionnaire (SFFQ) validated for the Mexican population was implemented (20). This tool consisted of 161 items inquiring about the average consumption of specific foods over the previous year. The SFFQ was administered only at baseline to characterize habitual intake; it was not reapplied during the follow-up period as dietary patterns were assumed to remain stable in these adult participants. This choice is justified by the study's non-interventional nature regarding nutrition, in which no lifestyle modifications were prescribed, and by the fact that the non-periodontitis group was only scheduled for a single initial visit. The SFFQ utilized a broad nine-option scale for intake frequency, ranging from ‘never’ to ‘more than six servings per day’. Frequency data were converted into grams or milliliters by multiplying standard portions by the reported portion size and normalizing the result to daily values (e.g., dividing by 30 for monthly intake or by 7 for weekly intake). For example, a standard 200 mL milk serving consumed three times per week resulted in a daily intake of 85.7 g [(200 × 3)/7]. To enhance precision, a photographic album of Mexican foods was used as a visual aid to estimate portions (21). Vegetable nitrate intake was calculated from the SFFQ data and established reference concentrations per 100 grams of specific vegetables (22). Dietary components previously associated with systemic oxidative and nitrosative status were prioritized for further analysis (23), with nutrient density expressed per 1000 kcal. Final calculations for energy and nutrient intake were performed using Nutritionist Pro™ software version 8.1.0 (Axxya Systems, Stafford, TX, USA).

### 2.6 Anthropometric and blood pressure assessment

All anthropometric measurements followed the protocol of the International Society for the Advancement of Kinanthropometry ISAK (24); weight, height, and waist circumference were taken. For weight measurement, the TANITA model HD-366 scale was used. Height was measured using a SECA 213 stadiometer, and hip circumference was measured with a Lufkin W606PM tape. For blood pressure measurement, the patient rested for 15 min before the measurements began. An OMRON HEM-742 digital blood pressure monitor was used; three repetitions of measurements were taken in a row on both arms (left and right) with 5 min of rest between each repetition (25).

### 2.7 Blood samples and biochemical analyses

Blood samples for serum and plasma analysis (5 mL) were simultaneously collected from the cubital vein into vials containing EDTA and without it, and were centrifuged at 1200× g at room temperature for 15 min. Serum and plasma samples were stored at −80 °C. Serum samples were analyzed using the VITROS 350 dry chemistry equipment, which measured the following analytes: glucose, triglycerides, total cholesterol, high-density lipoproteins (HDL), low-density lipoproteins (LDL), and very low-density lipoproteins (VLDL).

### 2.8 Unstimulated saliva sampling and pH quantification

To obtain the saliva sample, a 15 mL tube was given to the patient to deposit the accumulated saliva for 5 min. Participants were instructed to refrain from eating, drinking, and performing oral hygiene procedures for 12 h before saliva collection. Unstimulated whole saliva was collected from all patients by expectoration into sterile bulbs. Collected samples were immediately placed on ice and transported to the laboratory, where they were centrifuged at 5000 rpm for 10 min. The clear supernatants were stored in aliquots at −80 °C. For pH measurements, the Reflectoquant RQflex 10 reflectometer (Merck Millipore, Burlington, Massachusetts, USA) was used. This method is based on the intensity of light reflected by two reagent pads on test strips that change color intensity as a function of the concentration of a specific substance (26). The reagent strips (Reflectoquant, Merck Millipore) for pH had a range of pH 4–9. This was described by Rosier et al. 2020 (27).

### 2.9 Quantification of lipid peroxidation products

The levels of lipid peroxidation (LPO) were assessed using a commercially available kit (FR12; Oxford Biomedical Research, Oxford, MI, USA) according to the manufacturer's procedure. The method is based on the interaction between N-methyl-2-phenylindole, a chromogenic reagent, with malondialdehyde (MDA) and 4-hydroxyalkenals (4-HDA). Calibration curves were created using 1,1,3,3-tetramethoxypropane standards prepared in the Tris-HCl buffer. To perform the assay, 200 μL of saliva and plasma were combined with 455 μL of N-methyl-2-phenylindole in acetonitrile (Reagent 1), pre-diluted with ferric acid in methanol. After thorough mixing, 105 μL of methanesulfonic acid was added, and the mixture was incubated for 60 min at 45 °C. The samples were then centrifuged at 15,000 g for 10 min at room temperature. A 200 μL aliquot of the supernatant was transferred to a microplate, and its absorbance was recorded at 586 nm (23).

### 2.10 Nitric oxide catabolites (nitrate/nitrite)

To quantify nitric oxide (NO) catabolites, plasma and saliva samples were deproteinized by adding 6 mg of zinc sulfate to 400 μL of each sample, followed by agitation for 1 min and centrifugation at 10,000 × g for 10 min at 4 °C. The supernatant was collected and stored at −80 °C. NO catabolite levels were measured using a colorimetric assay (Nitric Oxide Assay Kit, user protocol 482650; Calbiochem®, San Diego, CA, USA) following the manufacturer's procedure. Eighty-five μL of standard or sample was placed in a well, and 10 μL of nitrate reductase and 10 μL of 2 mM NADH were added. The plate was agitated for 20 min at room temperature. Then, 50 μL of colorant was added and agitated, followed by the addition of 50 μL of colorant 2, which was stirred at room temperature. The plate was then read at 540 nm (23).

### 2.11 Total antioxidant capacity

Total antioxidant capacity (TAC) was measured following the manufacturer's protocol (Total Antioxidant Power Kit, No. TA02.090130, Oxford Biomedical Research). Standards and samples (saliva and plasma) were diluted 1:40 with the dilution buffer provided in the kit, and 200 μL of the prepared solution was dispensed into each well of a microplate. The initial absorbance of 450 nm was recorded as a reference value. Subsequently, 50 μL of copper solution was added, and the samples were incubated for 3 min at room temperature. Following incubation, 50 μL of stop solution was added, and the absorbance was measured again at 450 nm. TAC results were expressed as μM Trolox equivalents (28).

### 2.12 Carbonyl groups in proteins

A total of 200 µL of plasma and saliva samples was mixed with 500 µL of 10 mM 2.4-dinitrophenylhydrazine dissolved in 2 M HCl and incubated for 1 h at room temperature. Subsequently, 200 µL of 30% trichloroacetic acid was added, and the samples were centrifuged for 20 min at 14,000 × g. The resulting pellets were washed three times with 1 mL of an ethyl acetate-ethanol mixture (1:1, v/v). Following this, 600 µL of 6 M guanidine hydrochloride was added to the pellets, and the mixture was incubated for 15 min at room temperature. The absorbance was then measured at 370 nm (28).

### 2.13 Statistical analysis

Statistical analysis was performed using IBM SPSS® Statistics 25.0 IOS and RStudio Version 2024.12.0. Data analyses were conducted for all participants according to the defined groups. Normality was assessed by using the Shapiro–Wilk test. We expressed the results using medians and interquartile range. Fisher's exact test and Pearson's Chi square were used to compare categorical outcomes. Mann–Whitney U tests were used for between-group comparisons, while the Wilcoxon signed-rank test was used for paired-sample analysis to compare pre and post-treatment values within the periodontitis group. Kendall's Tau b was used to analyze association between groups for BMI distribution. Spearman's test was used for correlations. A *p*-value < 0.05 was considered statistically significant.

## 3 Results

### 3.1 General characteristics and biochemical measurements of participants

Figure 1 shows the flowchart of the study participants, and Table 1 shows the sociodemographic and anthropometric characteristics of our patients, while Table 2 presents biochemical measurements in both groups. There were no significant differences between groups.

**Table 1:** General and anthropometric characteristics of the examined groups.

Variable	Periodontitis (*n* = 11)	Non-periodontitis (*n* = 13)	*p*
Age, years	49.0 (19.0)	41.0 (8.0)	0.098^a^
Sex, F/M [% F]	9/2 [81.8]	6/7 [46.2]	0.105^b^
Weight, kg	68.90 (22.10)	70.10 (33.05)	0.451^a^
Height, m	1.56 (0.14)	1.69 (0.15)	**0**.**027**^a^
BMI, kg/m^2^	26.53 (7.52)	24.25 (7.57)	0.582^a^
Normal	4 (36.4)	7 (53.8)	0.681^c^
Overweight	5 (45.5)	4 (30.8)	0.454^d^
Obesity I	2 (18.2)	2 (15.4)	
WC, cm	84.00 (12.20)	82.50 (24.25)	0.885^a^
SBP, mmHg	118.00 (14.00)	117.70 (27.00)	0.977^a^
DBP, mmHg	75.00 (12.00)	72.80 (12.00)	0.685^a^

Values medians and interquartile range (IQR) for quantitative variables or *n* [%] qualitative variables. BMI; body mass index, cm; centimeters, DBP; diastolic blood pressure, F; female, kg; kilograms, M; male, m; meters, SBP; systolic blood pressure, WC; waist circumference.

Significant *p* value < 0.05.

a Mann–Whitney test.

b Fisheŕs exact test.

c Pearson's Chi square.

d Kendall's Tau b.

**Table 2:** Biochemical characteristics of the population.

Variable	Periodontitis (*n* = 11)	Non- Periodontitis (*n* = 13)	*p*
Glucose, mg/dL	104.00 (22.00)	102.00 (21.00)	0.954
Cholesterol, mg/dL	210.00 (58.00)	219.00 (101.00)	0.469
Triglycerides, mg/dL	211.00 (132.00)	153.00 (117.00)	0.311
HDL, mg/dL	44.00 (10.00)	46.00 (34.00)	0.602
LDL, mg/dL	124.00 (36.00)	135.00 (70.00)	0.417
VLDL, mg/dL	42.00 (26.00)	31.00 (24.00)	0.338

Values median and (IQR).

dL; deciliter, HDL; high density lipoprotein, LDL; low-density lipoprotein, mg; milligrams, VDLC; very low-density lipoprotein. Mann–Whitney test. Significant *p* value < 0.05 .

### 3.2 Periodontal evaluation of patients with and without periodontitis participants

All our patients were classified as stage III according to the 2017 Workshop. One patient (9.1%) was classified as grade A, five patients (45.5%) as grade B, and five patients (45.5%) as grade C.

Table 3 presents the periodontal clinical variables and pH measures for the two groups. A comparative analysis of salivary flow revealed no statistically significant differences between the groups. When comparing the periodontal values of patients with and without periodontitis after periodontal treatment, we found that statistical differences persist, without reaching values comparable to those of their counterparts without periodontitis. Likewise, when comparing the final salivary pH values in patients with periodontitis, we found a statistically significant difference compared to the non-periodontitis group. Furthermore, when comparing the periodontal clinical parameters in the periodontitis group, significant changes were observed after NSPT in PPD, and BoP, except for CAL and PoC. Following NSPT, the periodontitis group showed highly robust clinical improvements (*p* < 0.01) in several parameters. Specifically, significant reductions were observed in probing pocket depth (PPD), bleeding on probing (BOP), and the percentage of 4–5 mm pockets (*p* = 0.003).

**Table 3:** Periodontal clinical variables and saliva measures.

Variable	Non-periodontitis (*n* = 13)	Periodontitis pre-Tx (*n* = 11)	p^c^	Periodontitis post-Tx (*n* = 11)	p^d^	p^e^
Salivary flow, mL/min	0.40 (0.40)	0.40 (0.07)	0.904^a^	0.40 (0.25)	0.735^b^	0.770^a^
Salivary pH	7.50 (0.50)	7.10 (0.70)	0.067^a^	7.20 (0.50)	0.812^b^	**0.011** ^a^
PPD, mm	2.32 (0.49)	3.51 (0.83)	**<0.0001** ^a^	2.70 (0.72)	**0**.**006**^b^	**0.034** ^a^
CAL, mm	0.03 (0.30)	3.25 (1.24)	**<0.0001** ^a^	2.82 (1.22)	0.091^b^	**<0.001** ^a^
BOP, %	9.00 (9.99)	74.00 (40.00)	**<0.0001** ^a^	39.00 (19.00)	**0**.**023**^b^	**<0.001** ^a^
PoC, %	6.00 (7.15)	44.00 (60.00)	**<0.0001** ^a^	30.00 (36.00)	0.059^b^	**<0.001** ^a^
%PPD 4–5 mm	NA	30.30 (18.00)	NA	12.00 (9.00)	**0**.**003**^b^	NA
%PPD 4–5 + BOP	NA	23.00 (17.00)	NA	8.20 (5.00)	**0**.**008**^b^	NA
%PPD >6 mm	NA	5.00 (5.80)	NA	1.00 (2.12)	**0**.**023**^b^	NA
%PPD >6 mm + BOP	NA	2.00 (4.80)	NA	0.60 (1.28)	**0**.**033**^b^	NA

Values medians and (IQR).

PPD: periodontal probing depth, CAL: clinical attachment loss, BOP: bleeding on probing, PoC: Plaque or Dental Calculus, mm: millimeter, Tx: treatment, NA: no ability.

Significant *p* value < 0.05.

a Mann–Whitney test.

b Wilcoxon test.

c Comparison between non-periodontitis and pre-Tx periodontitis.

d Comparison between pre-Tx and post-Tx.

e Comparison between healthy and post-Tx.

### 3.3 Dietary intake with and without periodontitis participants

To further contextualize these findings, the daily intake was evaluated relative to international Recommended Dietary Allowances (RDA). This analysis clarifies that the significantly higher energy intake (*p* = 0.040) and lower PUFA consumption (*p* = 0.030) in the periodontitis group represent metabolic excesses and absolute deficiencies, respectively, relative to physiological requirements, providing a deeper clinical perspective than group comparison alone.

Table 4 shows the intake of the macronutrients and micronutrients. It was observed that patients with periodontitis consumed significantly more kilocalories (39% more than non-periodontitis group), and relatively fewer polyunsaturated fatty acids (PUFA) (29% less compared to participants without periodontitis). Supplementary Table S2 shows the correlations between all dietary components and oxidative/nitrosative stress parameters between groups, compared to participants without periodontitis.

**Table 4:** Comparison of daily nutrient intake volume between groups.

Variable	Periodontitis (*n* = 11)	Non-periodontitis (*n* = 13)	*p*
**Total energy, kcal/day**	**2071.3** (**1179.0)**	**1489.7** (**606.8)**	**0**.**040**
Carbohydrate, g/1,000 kcal	123.3 (28.6)	109.3 (33.28)	0.156
Lipid, g/1,000 kcal	40.8 (11.3)	43.7 (12.4)	0.099
Protein, g/1,000 kcal	39.6 (8.7)	42.9 (7.8)	0.839
Cholesterol, mg/1,000 kcal	173.3 (58.2)	156.9 (65.2)	0.469
SFA, g/1,000 kcal	13.1 (2.6)	13.3 (3.0)	0.664
MUFA, g/1,000 kcal	14.7 (5.2)	18.2 (4.9)	0.068
**PUFA,g/1,000 kcal**	**7.1** (**2.8)**	**10.0** (**3.6)**	**0**.**030**
TFA, g/1,000 kcal	1.3 (0.7)	1.5 (0.6)	0.401
Linoleic acid, g/1,000 kcal	6.1 (2.7)	7.9 (3.3)	0.052
Linolenic acid, g/1,000 kcal	0.65 (0.1)	0.64 (0.3)	0.505
EPA, g/1,000 kcal	0.029 (0.02)	0.038 (0.07)	0.173
DHA, g/1,000 kcal	0.081 (0.07)	0.114 (0.18)	0.125
*α*-Tocopherol, mg/1,000 kcal	4.0 (1.4)	4.2 (2.6)	0.469
Vitamin C, mg/1,000 kcal	121.4 (96.5)	99.4 (61.2)	0.794
Zinc, mg/1,000 kcal	5.4 (1.0)	5.2 (1.2)	0.706
Copper, mg/1,000 kcal	0.9 (0.1)	1.0 (0.2)	0.060
Folate, μg/1,000 kcal	188.5 (102.3)	199.1 (66.1)	0.977
Dietary fiber, g/1,000 kcal	14.9 (3.9)	15.6 (4.6)	0.622
Total sugar, g/1,000 kcal	44.9 (22.1)	43.6 (10.8)	0.931
Vegetable nitrate, mg/day	70.6 (90.0)	77.2 (76.3)	0.839
Alcohol, g/1,000 kcal	0.9 (1.7)	2.0 (1.9)	0.235

Values medians and (IQR).

g; grams, kcal; kilocalories, mg; milligrams, mL; milliliter, NR; not recommended, MFA; monounsaturated fatty acids, PUFA; polyunsaturated fatty acids, SFA; saturated fatty acids, TC; total calories, TFA; trans fatty acids. EPA; eicosapentaenoic acid, DHA; docosahexaenoic acid. Mann–Whitney test. Significant *p* value < 0.05.

### 3.4 Oxidant and antioxidant parameters between groups

To fully understand the nitrosative, antioxidant, and oxidative status of patients with and without periodontitis, we evaluated the following parameters in both saliva and plasma before and after NSPT: TAC, LPO (MDA+4-HDA), nitrate/nitrite, and carbonyl groups in proteins. The results of these measurements are presented in Table 5, which shows that salivary LPO was significantly higher in the periodontitis group compared to the non-periodontitis group at baseline [0.75 [0.22] μM/L vs. 1.12 [0.47] μM/L, respectively, *p* = 0.002]. After NSPT, it reached levels comparable to those of periodontal health [0.75 [0.22] μM/L vs. 0.94 [0.49] μM/L, *p* = 0.147], but without a statistically significant difference from baseline (*p* = 0.333).

**Table 5:** Oxidant and antioxidant parameters between groups.

Variable	Non-periodontitis (*n* = 13)	Periodontitis pre-Tx (*n* = 11)	*p* ^a^	Periodontitis post-Tx (*n* = 11)	*p* ^b^	*p* ^c^
**Salivary parameters**						
MDA+4-HDA, μM/L	0.75 (0.22)	1.12 (0.47)	**0**.**002**	0.94 (0.49)	0.333	0.147
nitrate/nitrite, pmol/mL	8.45 (1.56)	8.25 (5.72)	0.706	7.77 (2.93)	0.594	0.401
TAC, μM/L	1.57 (0.78)	1.81 (0.78)	0.385	1.47 (0.79)	0.328	0.750
CARB, nmol/mL	4.97 (0.51)	5.38 (0.56)	0.068	5.18 (0.20)	0.120	0.339
**Plasma parameters**						
MDA+4-HDA, μM/L	1.12 (0.32)	1.08 (0.37)	0.582	1.02 (0.43)	0.929	0.487
nitrate/nitrite, pmol/mL	16.89 (11.37)	18.55 (12.25)	0.622	18.90 (10.12)	0.155	0.908
TAC, μM/L	2.47 (1.36)	1.79 (1.06)	**0**.**046**	2.33 (0.70)	**0**.**021**	0.931
CARB, nmol/mL	10.30 (1.17)	10.19 (1.62)	0.505	10.89 (1.81)	0.328	0.311

Values median and (IQR).

MDA; malondialdehyde, 4-HDA; 4-hydroxyalkenals, CARB; Carbonyl groups in proteins, TAC; total antioxidant capacity, pmol/mL; picomol/milliliter, Tx; treatment.

Significant *p* value < 0.05.

a Comparison between healthy and pre-Tx with Mann–Whitney test;.

b Comparison between pre-Tx and post-Tx with Wilcoxon test;.

c Comparison between healthy and post-Tx with Mann–Whitney test. .

Likewise, we found that patients without periodontitis have a higher plasma total antioxidant capacity compared to patients with periodontitis [2.47 [1.36] μM/L vs. 1.79 [1.06] μM/L, respectively, *p* = 0.046] and that after NSPT in patients with periodontitis, this parameter increases, making it comparable to their healthy control [2.47 [1.36] μM/L vs. 2.33 [0.70] μM/L, respectively, *p* = 0.931] and with a statistical difference concerning their baseline value before NSPT (*p* = 0.021).

**Figure 2:**
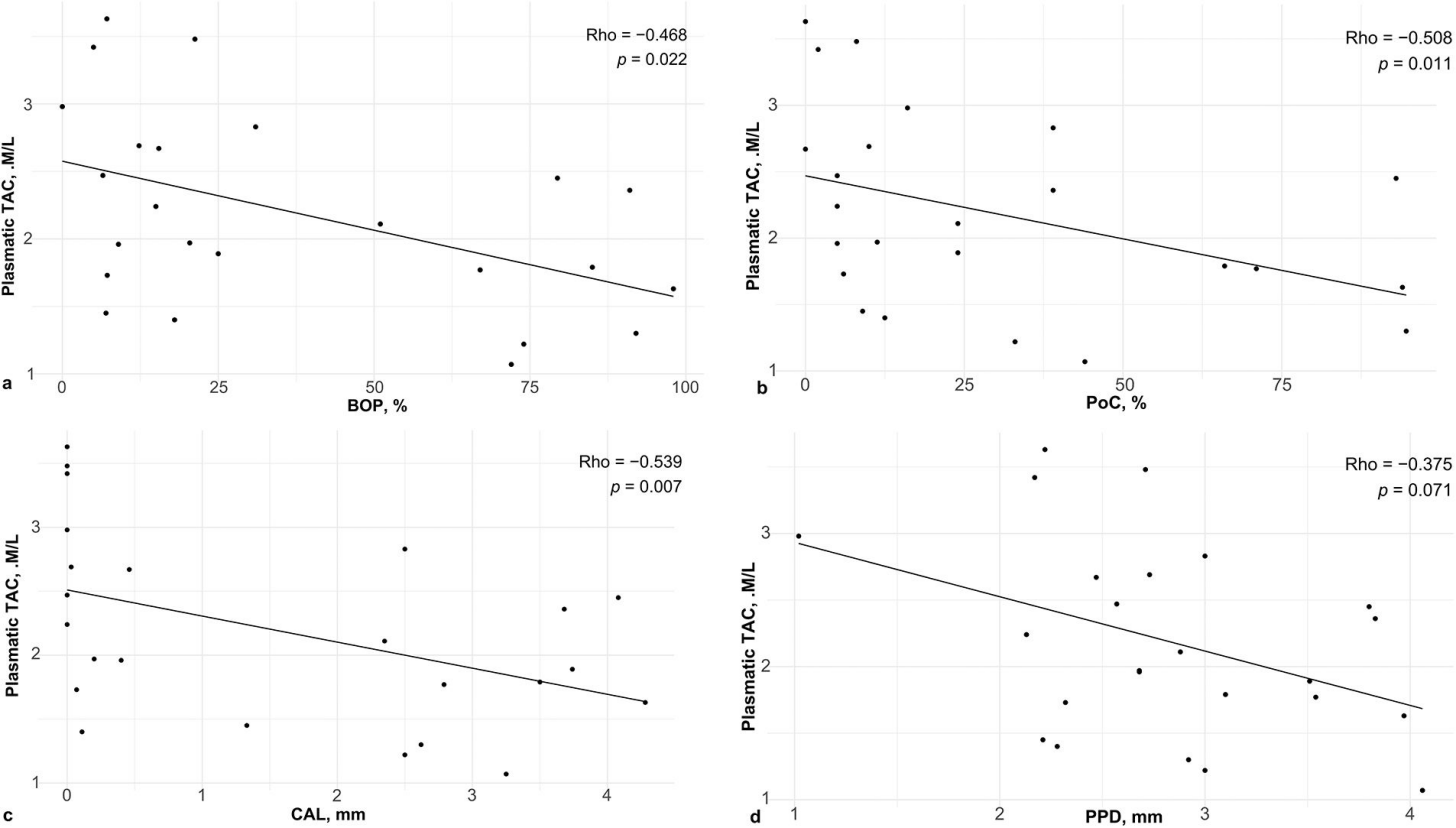
Correlations of TAC in plasma and periodontal clinical variables. (**a**) BOP, (**b**) PoC, (**c**) CAL and (**d**) PPD. TAC: total antioxidant capacity, BOP: bleeding on probing, PoC: Plaque or calculus, CAL: Clinical attachment loss. Spearman's correlation test. Significant *p* value < 0.05.

**Figure 3:**
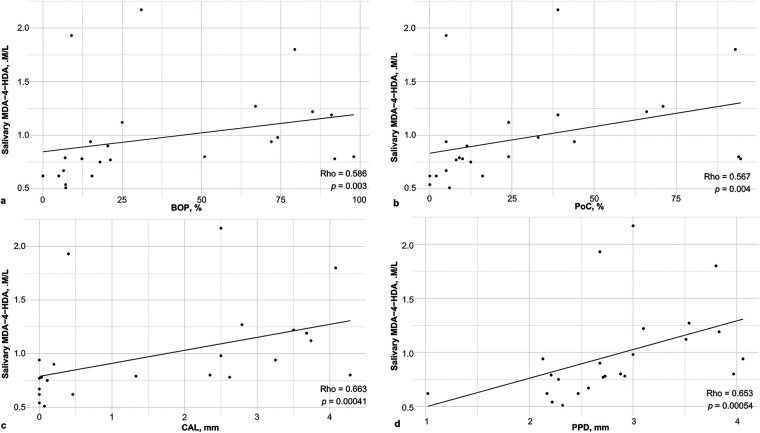
Correlations of LPO products and periodontal clinical variables. (**a**) BOP, (**b**) PoC, (**c**) CAL and (**d**) PPD. LPO: Lipoperoxidation, MDA: malondialdehyde, 4-HDA: 4-hydroxyalkenals, BOP: bleeding on probing, PoC: Plaque or calculus, CAL: Clinical attachment loss. Spearman's correlation test. Significant with *p* value < 0.05.

To determine the association between the oxidative/nitrosative status and clinical periodontal conditions, a correlation analysis was performed (Supplementary Tables S3, S4). The relationship between oxidative status and clinical parameters is presented in Figures 2, 3. Figure 2 highlights the negative correlation between plasmatic TAC and CAL (*p* = 0.007), while Figure 3 demonstrates the robust positive correlation between salivary LPO and all assessed periodontal variables.

We investigated whether nutrient intake had a relation with nitrosative/oxidative parameters by performing a correlation analysis between the selected (shown in Table 4) nutrient intake and these parameters. Table 6 shows the significant correlations of the dietary analysis of both samples. In the periodontal group we found a positive correlation between the intake of energy, vegetable nitrate, vitamin C, *α*-Tocopherol, total lipids, proteins, cholesterol, MUFA, PUFA, linolenic acid, zinc, cupper, folate and dietary fiber (*p* < 0.01), with the salivary nitrate/nitrite values. Furthermore, we found negative correlations between salivary MDA+4-HDA and energy, vitamin C, carbohydrates and dietary fiber and positive with alcohol (*p* < 0.01).

**Table 6:** Significant correlations between diet and oxidative/nitrosative parameters at baseline.

Periodontitis group (*n* = 11)				
Tissue	Oxidative parameters	Nutrient	Rho	*p*
**Saliva**	Nitrate/nitrite	Energy	0.700	0.016
		Lipid	0.636	0.035
		Protein	0.800	0.003
		Cholesterol	0.709	0.015
		MUFA	0.779	0.005
		PUFA	0.700	0.016
		Linolenic acid	0.609	0.047
		α-Tocopherol	0.700	0.016
		Vitamin C	0.891	<0.001
		Zinc	0.764	0.006
		Cupper	0.765	0.006
		Folate	0.655	0.029
		Dietary fiber	0.773	0.005
		Vegetable nitrate	0.700	0.016
	MDA+4-HDA	Energy	−0.615	0.044
		Carbohydrate	−0.743	0.009
		Vitamin C	−0.702	0.016
		Dietary fiber	−0.774	0.005
		Alcohol	0.633	0.036
**Plasma**	MDA+4-HDA	Lipid	−0.651	0.030
		Protein	−0.624	0.040
		Cholesterol	−0.761	0.007
		SFA	−0.621	0.042
		MUFA	−0.753	0.007
		PUFA	−0.620	0.042
		Linolenic acid	−0.606	0.048
		Vitamin C	−0.642	0.033
		Zinc	−0.615	0.044
	TAC	Vitamin C	−0.718	0.013
		Vegetable nitrate	−0.682	0.021
**Non-periodontitis group (*n*** **=** **13)**				
**Saliva**	TAC	TFA	−0.707	0.007
	MDA+4-HDA	Vitamin C	−0.569	0.042
**Plasma**	MDA+4-HDA	EPA	0.628	0.021
		DHA	0.620	0.024
		Vitamin C	0.561	0.046

MUFA; monounsaturated fatty acids, PUFA; polyunsaturated fatty acids, MDA; malondialdehyde, 4HDA; 4-hydroxyalkenals, CARB; Carbonyl groups in proteins, TAC; total antioxidant capacity, TFA; Trans Fatty Acids, SFA; Saturated Fatty Acids. Results of Spearman's correlation tests (rho) and *p* value of each component with salivary and plasmatic measurement.

Regarding plasma, it was found that MDA+4-HDA correlated negatively with protein, lipids, cholesterol, SFA, MUFAs, PUFAs, linolenic acid, vitamin C and zinc. Furthermore, TAC correlated negatively with vegetable nitrate and vitamin C.

In the non-periodontal group, in saliva we observed a negative correlation between TAC and trans fatty acids (TFA), and between MDA+4-HDA and vitamin C. In plasma, the following correlations were found significant; positive between MDA+4-HDA and vitamin C, EPA and DHA.

## 4 Discussion

This preliminary quasi-experimental study aimed to investigate the role of antioxidant components in the diet and non-surgical periodontal treatment (NSPT) in the oxidative, nitrosative, and antioxidant status of Mexican patients with stage III periodontitis. As expected, we observed improvements in both periodontal parameters and oxidative stress after NSPT. Notably, we found positive correlations between salivary MDA+4-HDA and all periodontal parameters, as well as between plasma TAC and all periodontal parameters. Regarding dietary intake, we found some differences between participants with and without periodontitis. Specifically, we observed that energy intake was higher in the periodontitis group, and PUFAs were ingested in lower proportions by those with the disease. Furthermore, we observed a positive correlation between salivary nitrate/nitrite concentrations and the intake of multiple dietary components, and negative correlations between salivary and plasma MDA+4-HDA and several diet components. Observational studies and systematic reviews (29, 30) have reported the association of diet with periodontitis. In turn, Wang et al. present a review of studies that have investigated the relationship between ROS and RNS secondary metabolites and periodontitis; however, to our knowledge, these correlations had not been reported in the population with periodontitis, even after our systematic searches.

Concerning clinical parameters, we observed significant improvements after treatment for all parameters except clinical attachment loss (CAL), which has been consistently reported in the literature in various populations, including the Mexican populations (31–34). Regarding oxidative stress parameters, we observed no differences in total antioxidant capacity in saliva; however, in blood, we found higher TAC in the non-periodontitis group and a significant increase after NSPT. Findings from a meta-analysis encompassing multiple population types revealed the same observation in the serum, plasma, and gingival crevicular fluid (GCF) of patients with periodontitis; however, this was not the case in saliva (35). Therefore, we consider that evaluating the total antioxidant capacity as an indicator of oxidative stress in a population with periodontitis should be performed in blood or GCF, not in saliva.

At local level, in saliva, specifically in the lipid peroxidation (LPO) indicators (MDA/4-HDA), we found differences between groups, with greater values in the subjects with periodontitis, those levels improved after therapy, in addition, MDA/4-HDA presented a positive correlation with periodontal parameters, this coincides with what has been observed by others (8, 36), and MDA has been proposed and reported as a diagnostic biomarker for periodontitis in different populations worldwide. Levels of MDA above 0.77 μmol/L are considered a cut-off point, as per Lorente's research (8). This is relevant since we observed a value of 0.75 μmol/L in subjects without periodontitis and values of 1.12 μmol/L in patients with the disease. Our findings indicate that energy intake in the periodontitis group exceeded the thresholds required to maintain physiological homeostasis, generating excessive substrates for mitochondrial respiration and accelerating ROS production. Although NSPT led to clinical improvements, it proved to be only a partial management measure; salivary MDA levels decreased to 0.94 μmol/L but remained above the 0.77 μmol/L healthy threshold (9). This suggests that mechanical biofilm removal alone is insufficient to fully restore oxidative homeostasis, as lipid peroxidation remains altered even after clinical recovery. This leads us to hypothesize that poor oral hygiene and the resulting persistent bacterial biofilm in patients with periodontitis acts as a continuous inflammatory stimulus, serving as a key contributing factor to the excessive local production of salivary MDA+4-HDA (8, 9).

It has been described that with age there may be a progressive deterioration in cellular and tissue functions due to oxidative stress (37). In our study, although we did not observe a significant difference in age between groups, patients in the periodontitis group were on average 8 years older. However, age did not correlate with oxidative/nitrosative stress variables except for salivary nitrates and nitrites, which did not show differences between the study groups (Supplementary Table S5).

As described above, NSPT does not appear to be enough to restore oxidative stress values comparable to those of subjects without periodontitis. It has been demonstrated that the antioxidant components of the diet influence the risk of developing periodontitis (38, 39). Therefore, we wondered if specific dietary components that have previously been shown to improve both oxidative and nitrosative stress status at the systemic level might have any relationship with these indicators (23). We observed that patients with periodontitis consumed more energy, consistent with enhanced cellular respiration (10, 11). In addition, the significantly lower intake of PUFA (*p* = 0.030) in the diseased group is critical, as these fatty acids possess a well-documented antioxidant capacity (12) that is notably deficient in this population, further compromising their ability to neutralize oxidative damage. However, the relationship between the intake of dietary antioxidant patterns and the risk of developing periodontitis has been demonstrated in extensive cohort studies (38, 39). Due to the nature of the studies, authors did not perform a detailed analysis of the periodontal clinical parameters, evaluate the levels of oxidative and/or nitrosative stress metabolites, nor delve into individual elements of the diet. On the other hand, Ashrafzadeh analyzed in more detail the components of the diet and the oxidative stress parameters TAC and MDA+4-HDA in the Iranian population; however, they did not include a control group without periodontitis, which did not allow to have a dietary reference value (40).

Furthermore, we sought to assess the relationship between oxidative and nitrosative parameters and dietary components before the NSPT. Overall, we found that the periodontal group presented more correlations than the non-periodontitis group (30 vs. 5). The most representative parameters were salivary nitrite/nitrates, with 14 positive correlations with dietary components in the periodontitis group (energy, vegetable nitrate, vitamin C, *α*-Tocopherol, total lipids, proteins, cholesterol, MUFA, PUFA, linoleic acid, zinc, cupper, folate and dietary fiber). Although we observed no differences between groups in nitrate/nitrite levels, others have reported an increase in the amount of nitrites in gingival crevicular fluid in patients with gingivitis and periodontitis compared to healthy subjects, but not in saliva (41). Nitric oxide (NO) metabolites have shown a duality in their participation in the pathophysiology of periodontitis, NO has antimicrobial activity and promotes the expression of extracellular matrix metalloproteinase 9, actively participating in the progression of the disease (42, 43) and, on the other hand, it has been proposed that nitrate functions as a probiotic for oral nitrate-reducing bacteria associated with oral health.

Additionally, we observed that MDA+4-HDA showed five significant correlations in saliva (negative correlations with energy, vitamin C, carbohydrates, and dietary fiber, and a positive correlation with alcohol intake) and nine in plasma (all negative: protein, total lipids, cholesterol, SFA, MUFA, PUFA, linolenic acid, vitamin C, and zinc). Notably, vitamin C was the only variable that showed overlapping significant correlations in both saliva and plasma. This unique interaction suggests that this vitamin acts as a pivotal bridge biomarker, facilitating communication between the systemic nutritional status and the local oral microenvironment. This relationship reinforces this potential as a key therapeutic target to modulate the body's antioxidant response in periodontal disease, opening the possibility that NSPT could be complemented with specific nutrient intake to achieve physiological balance.

Evidence regarding the relationship between nutrient intake and MDA levels has been reported in metabolic syndrome and pregnancy. A negative correlation has been observed between urinary MDA levels and adherence to the Mediterranean diet—characterized by high intakes of vitamins C and D, omega-3 fatty acids, and dietary fiber, and low saturated fat intake—in patients with metabolic syndrome (44). Similarly, during pregnancy, lower blood MDA levels have been associated with higher vitamin C intake (45), and supplementation with blueberries and fiber, (rich in antioxidants, including vitamin C) has also been shown to reduce plasma MDA (46, 47). Nevertheless, as far as we know, there are no reports linking nutrient intake with MDA levels in saliva or in periodontitis.

In the non-periodontitis group, we only observed two significant correlations in saliva (TAC with TFA; and MDA+4-HDA with vitamin C) and 3 in plasma (MDA+4-HDA with EPA, DHA and Vitamin C). We hypothesize that the lower frequency of correlations observed could be associated with a reduced oxidative stress status; consequently, the intake of antioxidant components may not be associated with the levels of these metabolites, as they do not appear to be altered.

The striking disparity in the number of correlations, 30 in the periodontitis group vs. 5 in the healthy control, provides a deeper insight into the biological state of the host. This difference suggests that in Stage III periodontitis, the homeostatic system is significantly stressed or sensitized, causing a metabolic hyper-reactivity, where oxidative and nitrosative markers become highly susceptible to fluctuations in dietary intake. While healthy individuals maintain a stable balance regardless of minor dietary changes, the diseased state exhibits a heightened metabolic sensitivity to nutritional antioxidant components. These results strengthen the hypothesis of a relationship between dietary antioxidant components and oxidative and nitrosative stress parameters in a population with periodontitis. As far as we know, this is the first study to demonstrate these correlations, which will need to be confirmed in future studies. They provide insight into these variables in stage III periodontitis patients, opening the possibility that NSPT could be accompanied by the intake of specific nutrients that modulate the body's antioxidant mechanisms, to achieve balance. To our knowledge, this is one of the few studies evaluating diet, oxidative and nitrosative stress parameters in Mexican subjects with stage III periodontitis. Additionally, the 2017 workshop classification was utilized to standardize the study population for greater homogeneity. The dietary collection tool is validated for the Mexican population.

The main limitation, besides those inherent to the study design, was the small number of patients included. Given the relatively small sample size and the poor oral hygiene throughout the study, our results should be considered preliminary and the conclusions interpreted with caution. While studies with small samples are valuable in the development of medical sciences (48). These studies strive for efficiency and more detailed measurements than those with large sample sizes. In this work, we carefully measured periodontal, dietary, and biomarker variables using validated methods and an interdisciplinary team specializing in periodontitis, nutrition, and oxidative stress. This is done to reduce confounding factors in the measurement of these variables, aspects that are not observed in this detail in studies with large sample sizes. Nevertheless, further studies such as cross-sectional, analytical follow-up, and randomized clinical trials are necessary to corroborate these findings with a larger sample and explore the molecular regulation mechanisms of these correlations.

## 5 Conclusions

In conclusion, this preliminary study suggests a direct relationship between dietary antioxidant intake and oxidative/nitrosative stress markers in Mexican patients with stage III periodontitis. While NSPT improves clinical parameters, the persistence of altered oxidative markers underscores the potential need for nutritional interventions to restore physiological homeostasis. These findings provide a basis for future large-scale randomized clinical trials to validate the efficacy of targeted dietary strategies in periodontal therapy.

## Data Availability

The original contributions presented in the study are included in the article/Supplementary Material, further inquiries can be directed to the corresponding author.
