# Supplementary material for: Interplay between dietary antioxidants and oxidative/nitrosative stress in stage III periodontitis: a preliminary quasi-experimental study in a Mexican population
Source: Front Oral Health. 2026 May 7;7:1779098. doi: 10.3389/froh.2026.1779098 (PMC13193204; doi:10.3389/froh.2026.1779098)
Supplement: Supplementary file 1 [file Table1.docx]

Supplementary Material

**Table S1.** CONSORT Statement for Randomized Trials of Nonpharmacologic Treatment

| Section/Topic | Item | Checklist item | # |
| --- | --- | --- | --- |
| Title and abstract | | | |
|  | 1a | Identification as a randomized trial in the title | N/A |
|  | 1b | Structured summary of trial design, methods, results, and conclusions (for specific guidance see CONSORT for abstracts) | 1-2 |
| Introduction | | | |
| Background and objectives | 2a | Scientific background and explanation of rationale | 2 |
|  | 2b | Specific objectives or hypotheses | 2-3 |
| Methods | | | |
| Trial design | 3a | Description of trial design (such as parallel, factorial) including allocation ratio | 3 |
|  | 3b | Important changes to methods after trial commencement (such as eligibility criteria), with reasons | 3-4 |
| Participants | 4a | Eligibility criteria for participants | 4 |
|  | 4b | Settings and locations where the data were collected | 3 |
| Interventions | 5 | The interventions for each group with sufficient details to allow replication, including how and when they were actually administered | 4 |
| Outcomes | 6a | Completely defined pre-specified primary and secondary outcome measures, including how and when they were assessed | 3-4 |
|  | 6b | Any changes to trial outcomes after the trial commenced, with reasons | N/A |
| Sample size | 7a | How sample size was determined | 3-4 |
|  | 7b | When applicable, explanation of any interim analyses and stopping guidelines | N/A |
| Randomization |  |  |  |
| Sequence generation | 8a | Method used to generate the random allocation sequence | N/A |
|  | 8b | Type of randomization; details of any restriction (such as blocking and block size) | N/A |
| Allocation concealment mechanism | 9 | Mechanism used to implement the random allocation sequence (such as sequentially numbered containers), describing any steps taken to conceal the sequence until interventions were assigned | N/A |
| Implementation | 10 | Who generated the random allocation sequence, who enrolled participants, and who assigned participants to interventions | N/A |
| Blinding | 11a | If done, who was blinded after assignment to interventions (for example, participants, care providers, those assessing outcomes) and how | N/A |
|  | 11b | If relevant, description of the similarity of interventions | N/A |
| Statistical methods | 12a | Statistical methods used to compare groups for primary and secondary outcomes | 7 |
|  | 12b | Methods for additional analyses, such as subgroup analyses and adjusted analyses | N/A |
| Results | | | |
| Participant flow (a diagram is strongly recommended) | 13a | For each group, the numbers of participants who were randomly assigned, received intended treatment, and were analyzed for the primary outcome | 3-4 |
|  | 13b | For each group, losses and exclusions after randomization, together with reasons | 3 |
| Recruitment | 14a | Dates defining the periods of recruitment and follow-up | N/A |
|  | 14b | Why the trial ended or was stopped | N/A |
| Baseline data | 15 | A table showing baseline demographic and clinical characteristics for each group | 7 |
| Numbers analyzed | 16 | For each group, number of participants (denominator) included in each analysis and whether the analysis was by original assigned groups | 7 |
| Outcomes and estimation | 17a | For each primary and secondary outcome, results for each group, and the estimated effect size and its precision (such as 95% confidence interval) | 7-8 |
|  | 17b | For binary outcomes, presentation of both absolute and relative effect sizes is recommended | N/A |
| Ancillary analyses | 18 | Results of any other analyses performed, including subgroup analyses and adjusted analyses, distinguishing pre-specified from exploratory | 7-8 |
| Harms | 19 | All important harms or unintended effects in each group (for specific guidance see CONSORT for harms) | N/A |
| Discussion | | | |
| Limitations | 20 | Trial limitations, addressing sources of potential bias, imprecision, and, if relevant, multiplicity of analyses | 11 |
| Generalizability | 21 | Generalizability (external validity, applicability) of the trial findings | 11 |
| Interpretation | 22 | Interpretation consistent with results, balancing benefits and harms, and considering other relevant evidence | 9-11 |
| Other information | | |  |
| Registration | 23 | Registration number and name of trial registry | 3 |
| Protocol | 24 | Where the full trial protocol can be accessed, if available | N/A |
| Funding | 25 | Sources of funding and other support (such as supply of drugs), role of funders | 12 |

| **Table S2.** Correlations between diet and oxidative/nitrosative parameters among groups | | | | | | | | | |
| --- | --- | --- | --- | --- | --- | --- | --- | --- | --- |
|  |  | **Nitrate/nitrite, pmol/ml (SL)** | **MDA-4-HDE, μM/L (SL)** | **TAC, μM/L (SL)** | **CARB, nmol/ml (SL)** | **Nitrate/nitrite, pmol/ml (PA)** | **MDA-4-HDE, μM/L (PA)** | **TAC, μM/L (PA)** | **CARB, nmol/ml (PA)** |
| **Periodontitis group (n= 11)** | | | | | | | | | |
| **Total energy** | Rho | **0.700^*^** | **-0.615^*^** | -0.173 | -0.273 | 0.282 | -0.569 | -0.464 | -0.374 |
|  | *p value* | **0.016** | **0.044** | 0.612 | 0.417 | 0.401 | 0.067 | 0.151 | 0.258 |
| **Carbohydrate** | Rho | 0.418 | **-0.743^**^** | -0.291 | -0.182 | 0.445 | -0.237 | -0.364 | -0.323 |
|  | *p value* | 0.201 | **0.009** | 0.385 | 0.593 | 0.170 | 0.483 | 0.272 | 0.332 |
| **Lipid** | Rho | **0.636^*^** | -0.296 | -0.009 | -0.373 | 0.100 | **-0.651^*^** | -0.245 | -0.264 |
|  | *p value* | **0.035** | 0.377 | 0.979 | 0.259 | 0.770 | **0.030** | 0.467 | 0.432 |
| **Protein** | Rho | **0.800^**^** | -0.474 | -0.118 | -0.309 | 0.200 | **-0.624^*^** | -0.509 | -0.323 |
|  | *p value* | **0.003** | 0.141 | 0.729 | 0.355 | 0.555 | **0.040** | 0.110 | 0.332 |
| **Cholesterol** | Rho | **0.709^*^** | -0.282 | -0.091 | -0.245 | 0.009 | **-0.761^**^** | -0.300 | -0.064 |
|  | *p value* | **0.015** | 0.400 | 0.790 | 0.467 | 0.979 | **0.007** | 0.370 | 0.852 |
| **SFA** | Rho | 0.473 | -0.369 | 0.045 | -0.073 | -0.045 | **-0.620^*^** | -0.055 | -0.305 |
|  | *p value* | 0.142 | 0.264 | 0.894 | 0.832 | 0.894 | **0.042** | 0.873 | 0.361 |
| **MUFA** | Rho | **0.779^**^** | -0.370 | -0.055 | -0.342 | 0.146 | **-0.753^**^** | -0.364 | -0.233 |
|  | *p value* | **0.005** | 0.263 | 0.873 | 0.304 | 0.669 | **0.007** | 0.270 | 0.491 |
| **Polyunsaturated** | Rho | **0.700^*^** | -0.301 | -0.082 | -0.400 | 0.100 | **-0.620^*^** | -0.391 | -0.251 |
|  | *p value* | **0.016** | 0.369 | 0.811 | 0.223 | 0.770 | **0.042** | 0.235 | 0.457 |
| **TFA** | Rho | 0.237 | 0.078 | -0.027 | -0.155 | -0.169 | -0.502 | 0.251 | 0.174 |
|  | *p value* | 0.483 | 0.821 | 0.936 | 0.649 | 0.620 | 0.115 | 0.457 | 0.610 |
| **Linoleic acid** | Rho | 0.600 | -0.223 | -0.118 | -0.482 | 0.100 | -0.547 | -0.255 | -0.292 |
|  | *p value* | 0.051 | 0.509 | 0.729 | 0.133 | 0.770 | 0.082 | 0.450 | 0.384 |
| **Linolenic acid** | Rho | **0.609^*^** | -0.355 | -0.045 | -0.364 | 0.136 | **-0.606^*^** | -0.218 | -0.323 |
|  | *p value* | **0.047** | 0.284 | 0.894 | 0.272 | 0.689 | **0.048** | 0.519 | 0.332 |
| **EPA** | Rho | 0.229 | -0.014 | 0.145 | -0.079 | -0.326 | -0.180 | -0.168 | -0.276 |
|  | *p value* | 0.499 | 0.967 | 0.671 | 0.817 | 0.327 | 0.596 | 0.622 | 0.412 |
| **DHA** | Rho | 0.129 | 0.055 | -0.032 | -0.179 | -0.317 | -0.161 | -0.032 | -0.207 |
|  | *p value* | 0.706 | 0.872 | 0.925 | 0.598 | 0.342 | 0.636 | 0.925 | 0.541 |
| **α-Tocopherol** | Rho | **0.700^*^** | -0.437 | -0.209 | -0.345 | 0.064 | -0.528 | -0.409 | -0.460 |
|  | *p value* | **0.016** | 0.179 | 0.537 | 0.298 | 0.853 | 0.095 | 0.212 | 0.154 |
| **Vitamin C** | Rho | **0.891^**^** | **-0.702^*^** | -0.136 | -0.109 | 0.473 | **-0.642^*^** | **-0.718^*^** | -0.105 |
|  | *p value* | **0.000** | **0.016** | 0.689 | 0.750 | 0.142 | **0.033** | **0.013** | 0.759 |
| **Zinc** | Rho | **0.764^**^** | -0.547 | -0.191 | -0.309 | 0.218 | **-0.615^*^** | -0.455 | -0.396 |
|  | *p value* | **0.006** | 0.082 | 0.574 | 0.355 | 0.519 | **0.044** | 0.160 | 0.228 |
| **Copper** | Rho | **0.765^**^** | -0.525 | -0.141 | -0.314 | 0.232 | -0.546 | -0.487 | -0.388 |
|  | *p value* | **0.006** | 0.097 | 0.679 | 0.346 | 0.492 | 0.083 | 0.128 | 0.238 |
| **Folate** | Rho | **0.655^*^** | -0.574 | -0.118 | -0.055 | 0.218 | -0.451 | -0.400 | -0.241 |
|  | *p value* | **0.029** | 0.065 | 0.729 | 0.873 | 0.519 | 0.164 | 0.223 | 0.474 |
| **Dietary fiber** | Rho | **0.773^**^** | **-0.774^**^** | -0.373 | -0.218 | 0.545 | -0.478 | -0.545 | -0.314 |
|  | *p value* | **0.005** | **0.005** | 0.259 | 0.519 | 0.083 | 0.137 | 0.083 | 0.346 |
| **Total sugar** | Rho | 0.400 | -0.574 | -0.173 | -0.155 | 0.091 | -0.260 | -0.255 | -0.524 |
|  | *p value* | 0.223 | 0.065 | 0.612 | 0.650 | 0.790 | 0.441 | 0.450 | 0.098 |
| **Vegetable nitrate** | Rho | **0.700^*^** | -0.355 | -0.182 | -0.218 | 0.218 | -0.396 | **-0.682^*^** | -0.278 |
|  | *p value* | **0.016** | 0.284 | 0.593 | 0.519 | 0.519 | 0.228 | **0.021** | 0.408 |
| **Alcohol** | Rho | -0.154 | **0.633^*^** | 0.520 | -0.124 | -0.530 | -0.166 | 0.466 | 0.065 |
|  | *p value* | 0.652 | **0.036** | 0.101 | 0.717 | 0.093 | 0.625 | 0.149 | 0.850 |
| **Non-periodontitis group (n= 13)** | | | | | | | | | |
| **Total energy** | Rho | -0.132 | -0.166 | -0.099 | -0.060 | 0.242 | 0.135 | 0.005 | -0.258 |
|  | *p value* | 0.668 | 0.588 | 0.748 | 0.845 | 0.426 | 0.661 | 0.986 | 0.394 |
| **Carbohydrate** | Rho | -0.082 | -0.298 | -0.027 | -0.044 | 0.096 | 0.091 | 0.027 | -0.253 |
|  | *p value* | 0.789 | 0.322 | 0.929 | 0.887 | 0.754 | 0.768 | 0.929 | 0.405 |
| **Lipid** | Rho | -0.302 | -0.055 | 0.110 | 0.170 | 0.446 | 0.022 | 0.225 | -0.093 |
|  | *p value* | 0.316 | 0.858 | 0.721 | 0.578 | 0.127 | 0.943 | 0.459 | 0.762 |
| **Protein** | Rho | -0.088 | 0.066 | -0.082 | -0.022 | 0.146 | 0.292 | 0.082 | -0.038 |
|  | *p value* | 0.775 | 0.830 | 0.789 | 0.943 | 0.635 | 0.334 | 0.789 | 0.901 |
| **Cholesterol** | Rho | -0.264 | 0.144 | -0.533 | 0.176 | -0.182 | 0.341 | 0.264 | 0.099 |
|  | *p value* | 0.384 | 0.640 | 0.061 | 0.566 | 0.553 | 0.254 | 0.384 | 0.748 |
| **SFA** | Rho | -0.479 | -0.014 | -0.041 | 0.215 | 0.200 | -0.096 | 0.259 | -0.088 |
|  | *p value* | 0.098 | 0.964 | 0.894 | 0.481 | 0.513 | 0.754 | 0.394 | 0.775 |
| **MUFA** | Rho | -0.346 | -0.276 | 0.291 | 0.357 | 0.435 | -0.036 | 0.308 | 0.011 |
|  | *p value* | 0.247 | 0.361 | 0.334 | 0.231 | 0.138 | 0.908 | 0.306 | 0.972 |
| **Polyunsaturated** | Rho | 0.121 | 0.077 | 0.088 | -0.148 | 0.432 | 0.195 | 0.049 | -0.192 |
|  | *p value* | 0.694 | 0.802 | 0.775 | 0.629 | 0.141 | 0.523 | 0.873 | 0.529 |
| **TFA** | Rho | 0.044 | 0.390 | **-0.707^**^** | 0.094 | -0.023 | 0.266 | 0.069 | 0.143 |
|  | *p value* | 0.886 | 0.188 | **0.007** | 0.761 | 0.939 | 0.380 | 0.823 | 0.641 |
| **Linoleic acid** | Rho | 0.049 | 0.099 | 0.115 | -0.137 | 0.421 | 0.074 | 0.071 | -0.214 |
|  | *p value* | 0.873 | 0.747 | 0.707 | 0.655 | 0.152 | 0.809 | 0.817 | 0.482 |
| **Linolenic acid** | Rho | 0.159 | 0.105 | 0.077 | -0.313 | 0.369 | 0.102 | -0.170 | -0.396 |
|  | *p value* | 0.603 | 0.733 | 0.803 | 0.297 | 0.215 | 0.741 | 0.578 | 0.181 |
| **EPA** | Rho | 0.312 | 0.205 | -0.342 | -0.025 | -0.294 | **0.628^*^** | -0.290 | 0.295 |
|  | *p value* | 0.300 | 0.501 | 0.253 | 0.936 | 0.329 | **0.021** | 0.337 | 0.328 |
| **DHA** | Rho | 0.265 | 0.178 | -0.331 | 0.044 | -0.288 | **0.620^*^** | -0.254 | 0.315 |
|  | *p value* | 0.381 | 0.561 | 0.269 | 0.886 | 0.341 | **0.024** | 0.402 | 0.295 |
| **α-Tocopherol** | Rho | 0.126 | 0.011 | 0.379 | 0.214 | 0.448 | -0.039 | 0.148 | -0.104 |
|  | *p value* | 0.681 | 0.971 | 0.201 | 0.482 | 0.124 | 0.901 | 0.629 | 0.734 |
| **Vitamin C** | Rho | 0.302 | **-0.569^*^** | -0.115 | 0.385 | 0.118 | **0.561^*^** | 0.258 | 0.060 |
|  | *p value* | 0.316 | **0.042** | 0.707 | 0.194 | 0.700 | **0.046** | 0.394 | 0.845 |
| **Zinc** | Rho | -0.036 | -0.149 | 0.165 | 0.061 | 0.289 | 0.127 | -0.055 | -0.195 |
|  | *p value* | 0.908 | 0.626 | 0.590 | 0.844 | 0.338 | 0.680 | 0.858 | 0.523 |
| **Copper** | Rho | 0.264 | -0.099 | -0.154 | -0.099 | 0.281 | 0.283 | -0.214 | -0.247 |
|  | *p value* | 0.384 | 0.747 | 0.616 | 0.748 | 0.353 | 0.348 | 0.482 | 0.415 |
| **Folate** | Rho | 0.170 | -0.166 | -0.242 | 0.214 | 0.011 | 0.410 | -0.011 | -0.170 |
|  | *p value* | 0.578 | 0.588 | 0.426 | 0.482 | 0.972 | 0.164 | 0.972 | 0.578 |
| **Dietary fiber** | Rho | 0.346 | -0.409 | 0.187 | 0.203 | 0.327 | 0.267 | -0.022 | -0.170 |
|  | *p value* | 0.247 | 0.165 | 0.541 | 0.505 | 0.275 | 0.378 | 0.943 | 0.578 |
| **Total sugar** | Rho | -0.209 | -0.160 | -0.341 | -0.060 | -0.085 | -0.129 | 0.005 | -0.137 |
|  | *p value* | 0.494 | 0.601 | 0.255 | 0.845 | 0.782 | 0.674 | 0.986 | 0.655 |
| **Vegetable nitrate** | Rho | 0.368 | -0.392 | -0.005 | 0.308 | 0.490 | 0.465 | 0.000 | -0.236 |
|  | *p value* | 0.216 | 0.185 | 0.986 | 0.306 | 0.089 | 0.109 | 1.000 | 0.437 |
| **Alcohol** | Rho | 0.298 | 0.233 | 0.315 | 0.204 | 0.094 | 0.534 | 0.149 | -0.094 |
|  | *p value* | 0.322 | 0.443 | 0.295 | 0.503 | 0.760 | 0.060 | 0.627 | 0.760 |
| MFA; monounsaturated fatty acids, PUFA; polyunsaturated fatty acids, SFA; saturated fatty acids, TFA; trans fatty acids. EPA; eicosapentaenoic acid, DHA; docosahexaenoic acid, MDA; malondialdehyde, 4HDA; 4-hydroxyalkenals, CARB; Carbonyl groups in proteins, TAC; total antioxidant capacity, PA; plasmatic sample, SL; salivary sample.  Results of Spearman's correlation tests (rho) and p value of each component with salivary and plasmatic measurement. | | | | | | | | | |

| **Table S3.** All correlations between periodontal and oxidative parameters (n = 24) | | | | | | | | | |
| --- | --- | --- | --- | --- | --- | --- | --- | --- | --- |
|  |  | **Nitrate/nitrite, pmol/ml (SL)** | **MDA-4-HDE, μM/L (SL)** | **TAC, μM/L (SL)** | **CARB, nmol/ml (SL)** | **Nitrate/nitrite, pmol/ml (PA)** | **MDA-4-HDE, μM/L (PA)** | **TAC, μM/L (PA)** | **CARB, nmol/ml (PA)** |
| **BOP** | Rho | 0.082 | **0.586**** | 0.114 | 0.192 | 0.155 | 0.000 | **-0.468*** | -0.220 |
|  | *p value* | 0.704 | **0.003** | 0.595 | 0.368 | 0.469 | 10.000 | **0.021** | 0.302 |
| **PoC** | Rho | 0.072 | **0.567**** | 0.184 | 0.182 | -0.017 | -0.245 | **-0.508*** | -0.229 |
|  | *p value* | 0.737 | **0.004** | 0.390 | 0.393 | 0.937 | 0.248 | **0.011** | 0.281 |
| **CAL** | Rho | 0.022 | **0.663**** | 0.158 | 0.066 | 0.137 | -0.255 | **-0.539**** | -0.130 |
|  | *p value* | 0.919 | **0.000** | 0.462 | 0.760 | 0.522 | 0.230 | **0.007** | 0.546 |
| **PPD** | Rho | 0.014 | **0.653**** | 0.123 | 0.203 | 0.237 | -0.187 | -0.375 | -0.171 |
|  | *p value* | 0.947 | **0.001** | 0.566 | 0.341 | 0.265 | 0.381 | 0.071 | 0.426 |
| PPD; periodontal probing depth, CAL; clinical attachment loss, BOP; bleeding on probing, PoC; Plaque or Dental Calculus, MDA; malondialdehyde, 4HDA; 4-hydroxyalkenals, CARB; Carbonyl groups in proteins, TAC; total antioxidant capacity, PA; plasmatic sample, SL; salivary sample.  Results of Spearman's correlation tests (rho) and p value of each component with salivary and plasmatic measurement. | | | | | | | | | |

| **Table S4.** Correlations between periodontal and oxidative parameters among groups | | | | | | | | | |
| --- | --- | --- | --- | --- | --- | --- | --- | --- | --- |
|  |  | **Nitrate/nitrite, pmol/ml (SL)** | **MDA-4-HDE, μM/L (SL)** | **TAC, μM/L (SL)** | **CARB, nmol/ml (SL)** | **Nitrate/nitrite, pmol/ml (PA)** | **MDA-4-HDE, μM/L (PA)** | **TAC, μM/L (PA)** | **CARB, nmol/ml (PA)** |
| **Periodontitis group (n= 11)** | | | | | | | | | |
| **BOP** | Rho | 0.145 | -0.364 | -0.200 | -0.082 | 0.109 | -0.018 | -0.300 | -0.328 |
|  | *p value* | 0.670 | 0.270 | 0.555 | 0.811 | 0.750 | 0.958 | 0.370 | 0.325 |
| **PoC** | Rho | 0.393 | -0.085 | 0.233 | -0.032 | -0.073 | -0.416 | -0.242 | -0.021 |
|  | *p value* | 0.232 | 0.805 | 0.491 | 0.926 | 0.831 | 0.203 | 0.473 | 0.952 |
| **CAL** | Rho | 0.246 | 0.119 | -0.314 | -0.524 | 0.264 | -0.395 | 0.077 | 0.005 |
|  | *p value* | 0.466 | 0.728 | 0.346 | 0.098 | 0.432 | 0.229 | 0.821 | 0.989 |
| **PPD** | Rho | 0.374 | 0.142 | -0.150 | -0.551 | 0.055 | -0.345 | -0.159 | -0.320 |
|  | *p value* | 0.258 | 0.678 | 0.659 | 0.079 | 0.873 | 0.299 | 0.640 | 0.338 |
| **Non-periodontitis group (n= 13)** | | | | | | | | | |
| **BOP** | Rho | 0.516 | 0.403 | 0.005 | -0.286 | 0.129 | 0.360 | -0.176 | -0.099 |
|  | *p value* | 0.071 | 0.172 | 0.986 | 0.344 | 0.674 | 0.226 | 0.566 | 0.748 |
| **PoC** | Rho | 0.177 | 0.261 | -0.069 | -0.426 | -0.263 | -0.231 | -0.362 | -0.396 |
|  | *p value* | 0.563 | 0.388 | 0.822 | 0.147 | 0.385 | 0.447 | 0.224 | 0.181 |
| **CAL** | Rho | 0.179 | 0.314 | 0.254 | **-0.618*** | -0.025 | -0.236 | **-0.685**** | -0.003 |
|  | *p value* | 0.558 | 0.296 | 0.402 | **0.024** | 0.936 | 0.438 | **0.010** | 0.993 |
| **PPD** | Rho | 0.011 | 0.293 | -0.014 | -0.096 | 0.426 | -0.079 | 0.003 | 0.085 |
|  | *p value* | 0.972 | 0.331 | 0.964 | 0.754 | 0.147 | 0.799 | 0.993 | 0.782 |
| PPD; periodontal probing depth, CAL; clinical attachment loss, BOP; bleeding on probing, PoC; Plaque or Dental Calculus, MDA; malondialdehyde, 4HDA; 4-hydroxyalkenals, CARB; Carbonyl groups in proteins, TAC; total antioxidant capacity, PA; plasmatic sample, SL; salivary sample.  Results of Spearman's correlation tests (rho) and p value of each component with salivary and plasmatic measurement. | | | | | | | | | |

| **Table S5.** Correlations between age, energy and oxidative and nitrosative stress parameters (n = 24) | | | | | | | | | | |
| --- | --- | --- | --- | --- | --- | --- | --- | --- | --- | --- |
|  |  | **Kcal** | **Nitrate/nitrite, pmol/ml (SL)** | **MDA-4-HDE, μM/L (SL)** | **TAC, μM/L (SL)** | **CARB, nmol/ml (SL)** | **Nitrate/nitrite, pmol/ml (PA)** | **MDA-4-HDE, μM/L (PA)** | **TAC, μM/L (PA)** | **CARB, nmol/ml (PA)** |
| **Age** | Rho | 0.049 | **-0.439*** | 0.208 | 0.119 | 0.310 | -0.096 | 0.333 | 0.114 | -0.039 |
|  | *p value* | 0.821 | **0.032** | 0.329 | 0.580 | 0.140 | 0.654 | 0.112 | 0.595 | 0.856 |
| MDA; malondialdehyde, 4HDA; 4-hydroxyalkenals, CARB; Carbonyl groups in proteins, TAC; total antioxidant capacity, PA; plasmatic sample, SL; salivary sample, Kcal; kilocalories. Results of Spearman's correlation tests (rho) and p value. | | | | | | | | | | |
